# HOCl‐Activated Aggregation of Gold Nanoparticles for Multimodality Therapy of Tumors

**DOI:** 10.1002/advs.202100074

**Published:** 2021-07-08

**Authors:** Dongya Liu, Lingyan Liu, Feiyang Liu, Mengfan Zhang, Peng Wei, Tao Yi

**Affiliations:** ^1^ Department of Chemistry Fudan University Shanghai 200438 China; ^2^ State Key Laboratory for Modification of Chemical Fibers and Polymer Materials College of Chemistry, Chemical Engineering and Biotechnology Donghua University Shanghai 201620 China

**Keywords:** gold nanoparticles, HOCl‐activated aggregation, photoacoustic imaging, photodynamic therapy, photothermal therapy

## Abstract

Tumor microenvironment‐responsive nanodrugs offer promising opportunities for imaging‐guided precision therapy with reduced side effects. Considering that the antitumor effect is closely related to the size of the nanodrugs, it is particularly important to develop a therapeutic system with size adjustability in the tumor microenvironment, which is still a great challenge in the field of nanotheranostics. Herein, a reactive oxygen species (ROS)‐activated aggregation strategy is reported for imaging‐guided precision therapy of tumors. The ROS‐activated nanoplatform is constructed based on gold nanoparticles (AuNPs) coated with an HOCl probe on its surface (namely, Au–MB–PEG NPs). The Au–MB–PEG NPs show high sensitivity toward HOCl, resulting in the modulation of surface charge and rapid aggregation of AuNPs, and simultaneous release of methylene blue as a photosensitizer for photodynamic therapy (PDT). In the tumor environment, the aggregated AuNPs ensure higher tumor accumulation and retention. Furthermore, the redshift of the absorption of aggregated AuNPs leads to activated photoacoustic imaging signals and photothermal therapy (PTT) under near‐infrared irradiation. Au–MB–PEG NPs thus efficiently inhibit the tumor growth through combined PTT–PDT therapy. This work contributes to the design of stimuli‐induced size‐aggregation nanodrugs, thereby attaining advanced performance in cancer diagnosis and treatment.

## Graphical abstract

A reactive oxygen species (ROS)‐activated aggregation strategy is constructed based on gold nanoparticles (AuNPs) coated with an HOCl probe. The as‐prepared nanoreagent shows high sensitivity toward HOCl, resulting in rapid aggregation of AuNPs for activated photoacoustic imaging and photothermal therapy, and simultaneous release of methylene blue as a photosensitizer for photodynamic therapy. The HOCl‐activated theranostic platform thus attains advanced performance in cancer treatment.

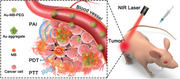

## 1 Introduction

Cancer is currently one of the most serious diseases that threatens human health and severely decreases the quality of life. Conventional chemotherapy often displays poor tumor specificity and suffers serious toxic side effects in cancer patients. Therefore, more accurate and effective treatment is urgently needed. More recently, tumor microenvironment (TME)‐responsive smart therapy systems have attracted increasing attention in the field of cancer therapy because they can take advantage of the difference between tumor and normal tissue microenvironments, thus decreasing side effects.^[^ ^1^, ^2^, ^3^, ^4^, ^5^, ^6^, ^7^, ^8^, ^9^, ^10^ ^]^ The TME exhibits several distinguishable physiological features such as mildly acidic pH, hypoxia, high level of reactive oxygen species (ROS), excess levels of GSH, and overexpression of specific enzymes.^[^ ^1^, ^2^, ^3^, ^4^, ^5^ ^]^ These features can be exploited as stimuli to induce specific changes in the chemical structures or physical properties of drugs to realize tumor‐specific treatment with less side effects.^[^ ^6^, ^7^, ^8^, ^9^, ^10^ ^]^

It is well‐known that nanodrugs can be passively delivered to tumor sites through enhanced permeability and penetration (EPR) effects. Using nanotechnology, various multifunctional nanodrugs have thus been developed for effective cancer therapies.^[^ ^11^, ^12^, ^13^, ^14^, ^15^ ^]^ However, the antitumor effect of the nanodrugs is closely related to their size.^[^ ^5^, ^10^, ^15^ ^]^ For example, large nanoparticles (>100 nm) are preferable, taking advantage of the EPR effect, but fail to fully infiltrate into tumor tissues due to the dense extracellular matrix and elevated interstitial fluid pressure of these tissues, thus leading to modest therapeutic benefits.^[^ ^16^, ^17^, ^18^ ^]^ Small nanoparticles (<20 nm) show much better tumor penetration capabilities and diffuse more uniformly within tumor tissues. However, they can be easily pumped back into the bloodstream and rapidly expelled from the body, resulting in insufficient tumor accumulation.^[^ ^19^, ^20^, ^21^ ^]^ It is therefore particularly important to develop a therapy system with size adjustability in the TME.

The general strategy for constructing a TME‐responsive therapy is to combine functional materials or drugs into a TME‐responsive nanocage, which can be dissociated by the TME to uncage the drugs, resulting in size shrinkage of the nanodrugs.^[^ ^15^, ^22^ ^]^ This will cause insufficient tumor accumulation due to the rapid elimination of small drugs from the tumor tissue. Stimuli‐induced size aggregation systems, which can fully utilize the advantages of nanoparticles, are therefore more effective. Unfortunately, the present reported stimuli‐responsive aggregation systems are very slow (Table S1, Supporting Information). The sensitivities and response times with stimuli are a key factor in designing size‐tunable nanoparticles. If the reaction time of nanoparticles is too long, a majority of the original small nanoparticles may be eliminated before aggregation occurs.^[^ ^16^ ^]^ It is therefore important to construct an aggregation system, which can be quickly activated by active substances highly expressed in tumor regions. The high expression of ROS is a typical characteristic of solid tumors, which can be used as a trigger to activate antitumor therapy.^[^ ^23^, ^24^, ^25^, ^26^, ^27^, ^28^ ^]^ ROS has been widely used as a typical stimulus in size shrinkage strategies for cancer therapy, although high ROS concentrations (typically >100 × 10^−6^ m) are needed to ensure their efficacy.^[^ ^29^, ^30^, ^31^ ^]^ It is therefore necessary to develop an aggregation strategy for nanoparticles, which is triggered by TME ROS in vivo with fast effects and high sensitivity.

**Scheme 1:**
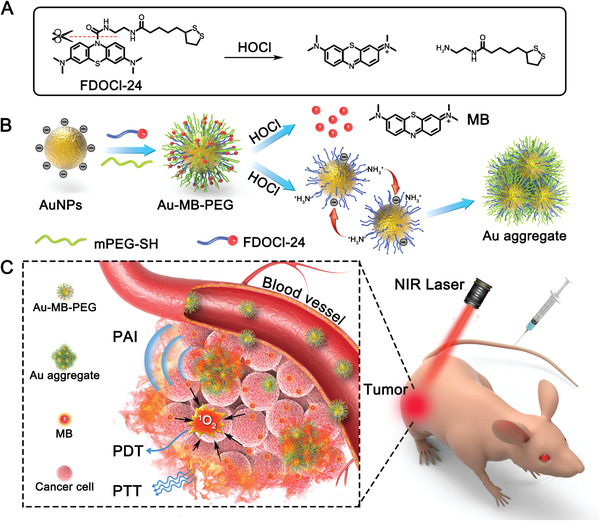
HOCl‐activated aggregation of AuNPs in vivo for enhanced PAI and combined PTT–PDT of tumor. A) Schematic reaction of HOCl‐responsive ligand FDOCl‐24. B) Illustration of synthesis of Au–MB–PEG and the mechanism of AuNPs aggregation. C) HOCl‐activated theranostic platform for enhanced PAI and combined PTT–PDT of tumor in vivo.

We have recently focused on ROS‐responsive systems. In particular, we have developed a platform, which can quickly release amino‐ or carboxy‐containing compounds triggered by HOCl for imaging and drug design,^[^ ^32^, ^33^, ^34^, ^35^, ^36^ ^]^ and further confirmed that the highly expressed HOCl in solid tumor areas was sufficiently active to uncage the fluorescent probe to release the fluorophores and functional groups.^[^ ^34^ ^]^ It is also known that the optical absorption of gold nanoparticles (AuNPs) can be redshifted to the near infrared (NIR) region by aggregation of small AuNPs into larger aggregates, resulting in photothermal therapy (PTT) and photoacoustic imaging (PAI) capabilities.^[^ ^37^, ^38^, ^39^ ^]^ Taking advantages of the excellent performance of both AuNPs and our HOCl‐responsive platform, we constructed a nanoagent by using AuNPs coordinated with an HOCl activatable ligand. Triggered by HOCl, the AuNPs could be aggregated with high efficiency for imaging‐guided combination therapy of tumors (**Scheme** **1**).

The HOCl‐responsive AuNPs were fabricated by attachment of PEG‐SH and an HOCl‐sensitive molecule (FDOCl‐24; Scheme 1A) on the nanoparticle surface by covalent Au─S bonds. Upon reaching tumor tissues, FDOCl‐24 molecules on the AuNPs were rapidly cleaved by HOCl to expose positive amino groups and release NIR emissive MB fluorophores. Driven by electrostatic interactions, the nanoparticles rapidly aggregated and accumulated in tumors, inducing redshift of the absorption, thus leading to enhanced PA imaging signals and PTT under laser irradiation (Scheme 1B,C). In addition, the released MB as a photosensitizer could be applied to PDT, to improve therapeutic efficacy through combination with PTT. This nanoplatform showed distinctive features for tumor theranostics, such as: 1) easy preparation compared to other nanotheranostic agents; 2) high sensitivity toward HOCl and rapid aggregation of AuNPs in the TME; and 3) enhanced PA imaging and tumor inhibition via the combination of PTT and PDT.

## 2 Results and Discussion

### 2.1 Design and Characterization of HOCl‐Responsive Au–MB–PEG NPs

Citrate‐capped AuNPs with a negatively charged surface (≈13 nm) were synthesized using a similar previously described method (Figure S1, Supporting Information).^[^ ^40^ ^]^ We regulated the aggregation of AuNPs by modulating the surface charge of the nanoparticles, because amino groups can combine with H^+^ ionized by water to form a positively charged state. FDOCl‐24, which released amino group upon the addition of HOCl, was designed and synthesized according to our previous report,^[^ ^26^ ^]^ and was conjugated onto the surface of AuNPs by covalent Au─S bonds (Figures S2–S7, Supporting Information). PEG‐SH (molecular weight: 5000) was then attached onto the AuNPs to prevent nonspecific aggregation and improve the stability of nanoparticles (namely, Au–MB–PEG). For comparison, AuNPs coated only with PEG‐SH were also prepared as a control of the HOCl‐insert AuNPs (Au–PEG).

The characterization of the constructed nanoparticles is shown in Figure S8 in the Supporting Information. The hydrodynamic diameters of AuNPs, Au–PEG NPs, and Au–MB–PEG NPs were 22, 30, and 45 nm, respectively, indicating the successful attachment of surface molecules on the AuNPs (Figure S8A, Supporting Information). The negative charge of AuNPs was significantly reduced due to the substitution of citrate by FDOCl‐24 and PEG‐SH (Figure S8B, Supporting Information). Furthermore, Fourier‐transform infrared analysis also confirmed the surface conjugation of AuNPs, where the strong bands at 1650 and 1108 cm^−1^ were attributed to benzene ring bending vibrations in the FDOCl‐24, and ether bonds of PEG chains were observed in the IR spectrum of Au–MB–PEG NPs (Figure S8C, Supporting Information). In addition, Au–MB–PEG NPs were well‐dispersed in both water and Dulbecco's modified eagle medium supplemented with 10% fetal bovine serum without noticeable change in their UV–vis absorption, indicating the favorable physiological stability (Figure S9, Supporting Information). Although there was a small peak at about 670 nm from a little release of MB in culture medium after long‐time storage (Figure S9, Supporting Information), the degree of deterioration of FDOCl‐24 was very limited, which would not affect the response of Au–MB–PEG NPs toward HOCl (Figure S9B, Supporting Information).

**Figure 1:**
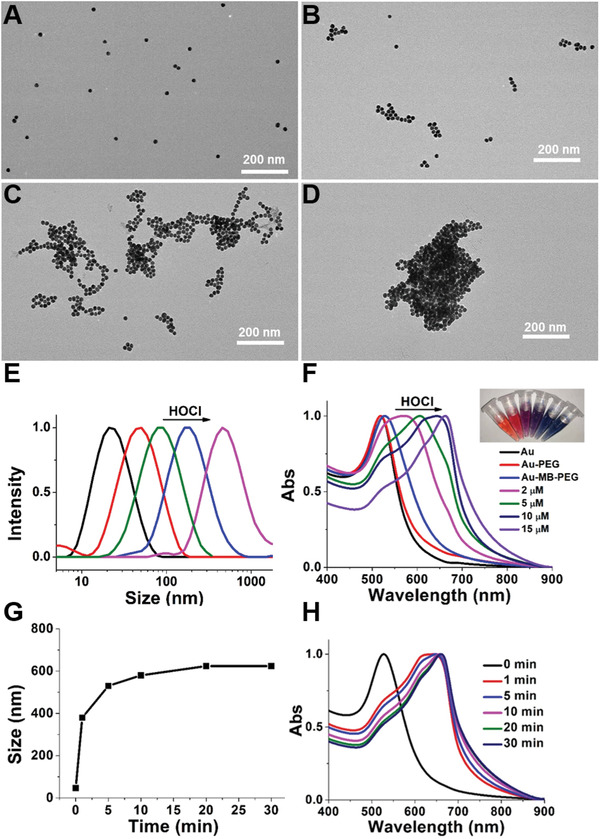
The characterization of Au–MB–PEG NPs. TEM images of A) Au–MB–PEG NPs only, B–D) with 2 × 10^−6^, 5 × 10^−6^,  and 10 × 10^−6^ m HOCl, respectively. E) Hydrodynamic diameter of AuNPs, Au–MB–PEG NPs, and Au–MB–PEG NPs in the presence of 2 × 10^−6^, 5 × 10^−6^, and 10 × 10^−6^ m HOCl from left to right. F) UV–vis absorption of Au NPs, Au–PEG NPs, and Au–MB–PEG NPs in the absence and presence of increasing concentration of HOCl; inset from left to right: AuNPs, Au–MB–PEG NPs, and Au–MB–PEG NPs with 2  × 10^−6^, 5 × 10^−6^, 10 × 10^−6^, and 15 × 10^−6^ m HOCl. Time evolutions of G) hydrodynamic size and H) absorption spectra for Au–MB–PEG NPs incubating with 10 × 10^−6^ m HOCl.

The as‐prepared Au–MB–PEG NPs showed good monodisperse morphology with the average size of about 20 nm (**Figure** **1**A). However, upon adding HOCl, the Au–MB–PEG NPs began to form particle aggregates due to the reaction between the FDOCl‐24 ligand and HOCl. As shown in Figure 1B–D and Figure S10 in the Supporting Information, after incubation with increasing concentrations of HOCl, Au–MB–PEG NPs gradually aggregated and simultaneously coupled with an increase of hydrodynamic diameter from 45 to 600 nm (Figure 1E). The absorption changes also verified the remarkable regulatory aggregation of Au–MB–PEG NPs. As shown in Figure 1F, the initial surface plasmon resonance peak of Au–MB–PEG NPs was at ≈528 nm, close to AuNPs and Au–PEG NPs at around 520 nm. However, the absorption maximum gradually shifted to ≈664 nm and extended to the NIR region of 700–900 nm upon the addition of HOCl, suggesting the release of MB and the aggregation of AuNPs. As shown in Figure S11 in the Supporting Information, in the superimposed absorption spectra of aggregated AuNPs and pure MB, the shadow area came from aggregation of AuNPs after subtracting of pure MB. At the same time, the color of Au–MB–PEG NP solutions changed from wine red to bluish gray because of both the production of MB and aggregation of AuNPs (Figure 1F, inset). This was also verified by fluorescence changes at 686 nm, shown in Figure S12A in the Supporting Information. Besides, FDOCl‐24 displayed good selectivity toward HOCl under the test conditions. Other ROS did not induce noticeable enhancement of the fluorescence intensity (Figure S12B, Supporting Information).

It should be noted that the reaction between Au–MB–PEG NPs and HOCl was very quick. The increase of hydrodynamic diameter took place within 1 min to reach a plateau in 10 min (Figure 1G) and the redshift of absorption showed similar results as in Figure 1H. These results indicated that Au–MB–PEG NPs showed high sensitivity toward HOCl with rapid aggregation of AuNPs in vitro.

Since HOCl could cleave FDOCl‐24 to produce positively charged amino groups, the aggregation mechanism of Au–MB–PEG NPs after response to HOCl might be the electrostatic interactions between positively charged amino groups and negatively charged carboxyl groups on different AuNPs. It is thus important to adjust the ratio of amino groups and carboxyl groups on one AuNP for interaction. Au–MB–PEG NPs with different amount of FDOCl‐24 were prepared with initial molar ratio of AuNPs and FDOCl‐24 varied from 1:1500 to 1:30 000. As shown in Figure S13 in the Supporting Information, the absorption of Au–MB–PEG NPs after reacting with the same concentration of HOCl (10 × 10^−6^ m) had a redshift with increasing adding amount of FDOCl‐24. Simultaneously, the corresponding hydrodynamic diameter of nanoparticles also gradually increased from 40 to 600 nm. The results demonstrated that the degree of aggregation was proportional to the adding amount of FDOCl‐24. It is obvious that insufficient FDOCl‐24 molecules decorated on the nanoparticles produce less positively charged amino groups, thus do not favor the aggregation of AuNPs. In the following experiment, we choose the ratio of 1:30 000 for the in vitro and in vivo tests. On the premise of producing adequate positively charged amino groups, we thus speculated that the bare amino groups of one nanoparticle could interact with original carboxyl groups of another nanoparticle by electrostatic interactions to lead to aggregation, as shown in Scheme 1. As the control group, transmission electron microscopy (TEM), UV–vis absorption spectra, and hydrodynamic diameter characterization of AuNPs coated by PEG only (Au–PEG) in the presence of HOCl were also conducted (Figures S14 and S15, Supporting Information). The results demonstrated that the AuNPs without FDOCl‐24 functionalization were nonresponsive to HOCl.

### 2.2 In Vitro Photothermal Performance, ROS Generation, and Photoacoustic Imaging of Au–MB–PEG NPs

Because Au–MB–PEG NPs displayed strong and broad NIR absorption (600–850 nm) with the release of MB photosensitizer upon the addition of HOCl, in vitro experiments were conducted to confirm the therapeutic capability of Au–MB–PEG NPs for treating cancer cells by using a combined PTT and PDT strategy. The photostability of Au–MB–PEG NPs was investigated by measuring the variations of dynamic light scattering and absorption spectra irradiated by 808 and 655 nm lasers for 30 min. As shown in Figure S16 in the Supporting Information, the absorption of Au–MB–PEG NPs showed negligible changes upon irradiation, but showed a redshift only when incubated with HOCl, indicating the good photostability and high HOCl sensitivity of Au–MB–PEG NPs. Therefore, the nanoparticles were used for further PTT and PDT applications.

**Figure 2:**
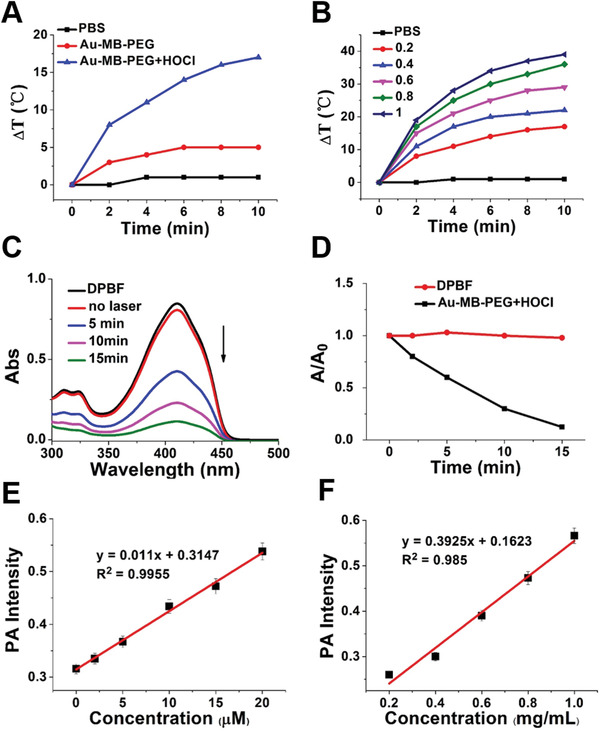
In vitro photothermal and photodynamic performance of Au–MB–PEG. Temperature changes of A) PBS, Au–MB–PEG NPs before and after treatment with 10 × 10^−6^ m HOCl and B) different concentrations of aggregated Au–MB–PEG NPs (from 0.2 to 1 mg mL^−1^, in the presence of 10  × 10^−6^ m HOCl) upon 808 nm laser irradiation (1.5 W cm^−2^, 10 min). C) UV–vis spectra of DPBF containing Au–MB–PEG NPs after being treated with 10 × 10^−6^ m HOCl for different irradiation times and D) corresponding relative absorbance variations under 655 nm laser irradiation (0.4 W cm^−2^). E) PA signal intensity of Au–MB–PEG NPs reacting with HOCl of different concentrations (these data are the mean ± s.d., *n* = 3 per group). F) PA signal intensity of aggregated Au–MB–PEG NPs at different concentrations from 0.2 to 1 mg mL^−1^ (these data are the mean ± s.d., *n* = 3 per group).

The photothermic behavior of Au–MB–PEG NPs was demonstrated using irradiation with an 808 nm laser (1.5 W cm^−2^). As shown in **Figure** **2**A, upon irradiation for 10 min, the temperature in Au–MB–PEG NPs (0.2 mg mL^−1^) containing solution increased by about 17 °C in the presence of 10 × 10^−6^ m HOCl. However, no significant temperature change was observed in the PBS control. The group containing Au–MB–PEG NPs without HOCl only showed a very limited temperature increase of 5 °C under the same conditions. The temperature increase had a positive correlation with the concentration of aggregates (from 0.2 to 1 mg mL^−1^), suggesting that Au–MB–PEG NPs had good photothermal performance after being activated by HOCl (Figure 2B).

The activatable photodynamic properties of Au–MB–PEG NPs were tested using 1,3‐diphenylisobenzofuran (DPBF) to detect ROS generation in vitro. In the presence of HOCl‐treated Au–MB–PEG NPs, the absorbance of DPBF at 410 nm rapidly decreased during 655 nm laser irradiation over 15 min, because of the generation of ^1^O_2_ from the released MB photosensitizer (Figure 2C,D). In addition, the absorption decrease of DPBF was strongly dependent on the concentration of HOCl from 2 × 10^−6^ to 10 × 10^−6^ m at the same irradiation time for 10 min (Figure S17, Supporting Information), indicating the excellent ^1^O_2_ generation ability of HOCl‐activated Au–MB–PEG NPs in vitro. The UV−Vis absorption spectrum of DPBF treated with 10 × 10^−6^ m HOCl as control showed no absorption change (Figure S18, Supporting Information), indicating that HOCl did not interfere with the ROS detection produced by PDT. These results suggested that Au–MB–PEG NPs simultaneously possessed HOCl‐activated photothermal performance and photodynamic properties.

As previously mentioned, the strong absorption band in the NIR region of aggregated Au–MB–PEG NPs facilitated the improvement of PTT efficacy and also increased the PA image signal. Figure 2E and Figure S19A in the Supporting Information show the HOCl concentration‐dependent PA signal intensity of Au–MB–PEG NPs. We then plotted the PA intensity as a function of HOCl concentration and found a good linear correlation, indicating the PA quantitation of HOCl in vitro. In addition, the PA signal of aggregated Au–MB–PEG NPs, obtained after the same HOCl concentration incubation, gradually increased with an increase of nanoparticle concentration (Figure 2F; Figure S19B, Supporting Information). Taken together, these results showed that the prepared Au–MB–PEG NPs were an HOCl‐responsive PAI contrast agent for tumor imaging.

### 2.3 In Vivo Photoacoustic and Photothermal Imaging of Au–MB–PEG NPs

**Figure 3:**
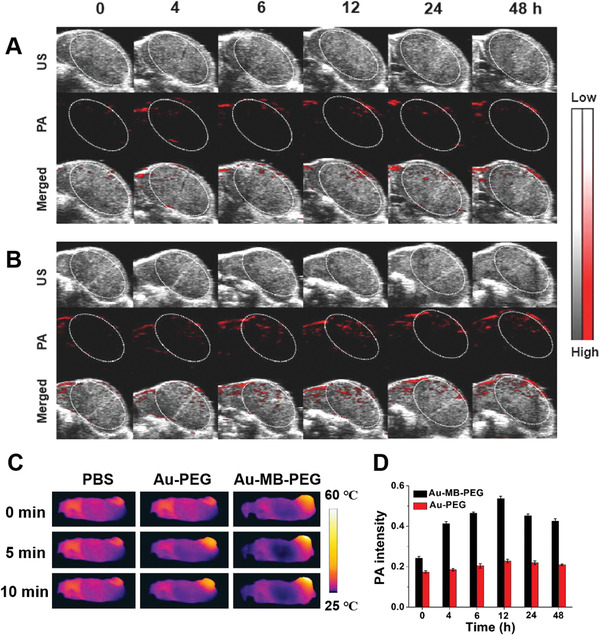
PA images of the tumor in HepG2 tumor‐bearing mice treated with A) Au–PEG NPs and B) Au–MB–PEG NPs. C) Photothermal images of tumor‐bearing mice for showing tumor local temperature against irradiation time of 808 nm laser after treatment with PBS, Au–PEG, and Au–MB–PEG NPs at 12 h postinjection. D) Corresponding quantified intensity of PA signal treated with Au–PEG NPs and Au–MB–PEG NPs. Values are the mean ± s.d. for *n* = 3 per group.

**Figure 4:**
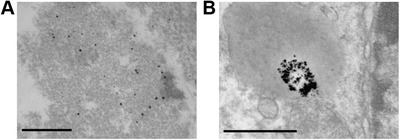
In vivo aggregation of Au–MB–PEG NPs. Bio‐TEM images of tumor tissues after injection of A) Au–PEG NPs and B) Au–MB–PEG NPs for 12 h. The scale bar is 500 nm.

The cytotoxicity of Au–MB–PEG NPs was first assessed using the methythiazolyl tetrazolium assay. As shown in Figure S20 in the Supporting Information, Au–MB–PEG NPs exhibited negligible cytotoxicity to HepG2 cells at a concentration range of 0.1–0.8 mg mL^−1^ after being incubated with cells for 12 and 24 h. The overall cell viability remained above 80%, suggesting the good biocompatibility of Au–MB–PEG NPs. To show the PAI activity in vivo, 200 µL of aqueous solution of Au–MB–PEG NPs (2 mg mL^−1^) was intravenously injected into the tail vein of living female nude mice bearing HepG2 tumors. As a control group, Au–PEG NPs with the same concentration were also injected. A weak signal change in the tumor site appeared within 48 h in the Au–PEG group (**Figure** **3**A). This result suggested that nonresponsive Au–PEG might be eliminated quickly due to the low accumulation in tumor tissue. In contrast, the PA signal in the tumor site, after being injected with Au–MB–PEG, gradually intensified from 4 to 6 h and reached a maximum at 12 h, indicating the efficient accumulation of AuNPs in tumor tissues (Figure 3B). The average tumor PA intensity of Au–MB–PEG NP‐treated mice was ≈2.5‐fold stronger than that observed from mice injected with Au–PEG NPs (Figure 3D). The enhanced tumor accumulation of Au–MB–PEG NPs could be ascribed to HOCl‐activated aggregation of AuNPs and thereby prolonged tumor retention. This was also confirmed by comparison of biotransmission electron microscopy images of tumor tissues treated with Au–PEG NPs and Au–MB–PEG NPs for 12 h (**Figure** **4**). Figure 4A,B shows the monodispersed and aggregated Au NPs in the tumor tissues, respectively. Because of these exciting results, the photothermal imaging of the Au–MB–PEG NPs was further investigated in vivo. As shown in Figure 3C and Figure S21 in the Supporting Information, the tumor local temperature was raised by 20 °C within 10 min of irradiation (808 nm, 1.5 W cm^−2^). In comparison, the local temperature increase in tumors was only 8 and 10 °C for PBS‐ and Au–PEG NP‐treated mice, respectively. Most importantly, the enhanced PA imaging and PT efficacy of Au–MB–PEG NPs could be ascribed to the larger particles formed via the self‐aggregation of Au–MB–PEG due to highly expressed HOCl levels in tumors, thus greatly enhancing the retention of AuNPs within the tumor.

### 2.4 The In Vivo Tumor Inhibitory Effect of Combined PTT–PDT

**Figure 5:**
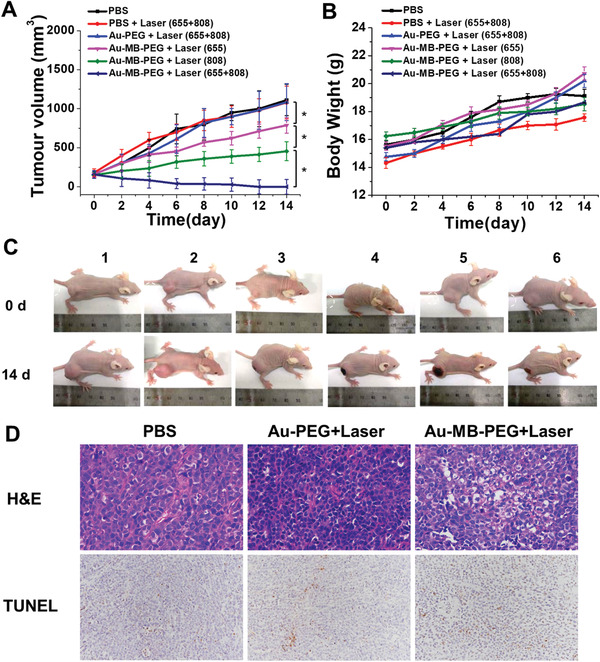
In vivo PTT–PDT therapy of tumor. A) Tumor growth curve and B) body weight variation of HepG2‐tumor bearing mice after different treatments. Values are the mean ± s.d. for *n* = 4, ^*^ *p* < 0.05 and ^**^ *p* < 0.01. C) Representative photos of mice after different treatments captured on prior treatment and 14th day post‐treatment. Groups 1–6: PBS, PBS+Laser (655+808), Au–PEG+Laser (655+808), Au–MB–PEG+Laser (655), Au–MB–PEG+Laser (808), and Au–MB–PEG+Laser (655+808). D) H&E and TUNEL staining in tumors after different treatments. The apoptosis rate from left to right in the TUNEL staining is 3.19%, 10.92%, and 30.88%, respectively.

The antitumor effects of Au–MB–PEG NPs were investigated using in vivo models. HepG2 tumor‐bearing BALB/c mice were randomly divided into six groups (four mice in each group): 1) injection with PBS as the control group; 2) injection with PBS followed by laser irradiation (laser, 808 + 655 nm); 3) injection with Au–PEG followed by laser irradiation (808 + 655 nm); 4) injection with Au–MB–PEG by PDT only (655 nm); 5) injection with Au–MB–PEG by PTT only (808 nm); and 6) injection with Au–MB–PEG followed by laser irradiation (laser, 808 + 655 nm). The photothermal and photodynamic treatments, irradiated with laser of 808 and 655 nm, respectively, were performed at 12 h postinjection, guided by in vivo PA imaging (Figure 3D). The therapy efficacy was evaluated by monitoring the average tumor size over a period of 14 days. As shown in **Figure** **5**A, the mice treated with PBS exhibited a rapid increase in tumor size. The group treated with HOCl‐inert Au–PEG under PDT and PTT showed no inhibition of tumor growth. Significantly, the tumors treated with Au–MB–PEG NPs using combinational PDT (from released MB) and PTT (from aggregated AuNPs) were completely eliminated, much more effectively than the groups treated with only PDT or PTT. Figure 5C and Figure S22 in the Supporting Information show representative photographs of mice and tumors harvested on the 14th day post‐treatment from mice after different treatments. The results indicated that Au–MB–PEG NPs exhibited synergistic PTT and PDT effects to efficiently inhibit tumor growth, which benefited from the HOCl‐triggered aggregation of AuNPs and release of MB from Au–MB–PEG NPs. Notably, negligible loss of body weight was observed for all groups of mice during the therapeutic period (Figure 5B). Moreover, as revealed by images of hematoxylin and eosin (H&E) staining and terminal deoxynucleotidyl transferase uridine triphosphate nick end‐labeling‐stained (TUNEL) tumor slices (Figure 5D), the most severe tumor cell damage was found in the last group, while cancer cells in the control groups were either not notably affected or only showed partial damage and apoptosis. Figure S23 in the Supporting Information shows that histological analyses of typical hearts, livers, spleens, lungs, and kidneys of the mice treated with Au–MB–PEG NPs using laser irradiation showing no significant damage to these organs, and indicating the excellent biocompatibility of the HOCl‐responsive nanoreagent. A blood biochemistry assay further verified the nontoxic nature of Au–MB–PEG NPs (Figure S24, Supporting Information).

## 3 Conclusion

In summary, we constructed a ROS‐activated aggregation system based on AuNPs coated with an HOCl‐responsive ligand. The original fabricated Au–MB–PEG NPs have suitable hydrodynamic diameters (≈45 nm) satisfied for both the EPR effect and capability of infiltrating into tumor tissues. The Au–MB–PEG NPs showed high sensitivity toward HOCl, resulting in rapid aggregation of AuNPs in HOCl high expressed tumor regions, and simultaneously releasing MB molecules. The activated aggregation platform thus exhibited good photothermal performance and enhanced PA imaging in vitro and in vivo. PDT could also be activated with the released MB photosensitizer. Thus, the tumor growth could be efficiently suppressed through combined PTT–PDT therapy in a tumor‐bearing mouse model. The construction of ROS‐responsive AuNPs provides a novel concept for the design of a stimuli‐responsive aggregation system. More importantly, this ROS‐activated aggregation strategy can potentially be extended into other material systems to achieve further development of nanomedicine for tumor theranostics.

## Conflict of Interest

The authors declare no conflict of interest.

## Supporting information

Supporting Information

## Data Availability

Research data are not shared.
