# Supplementary material for: HOCl‐Activated Aggregation of Gold Nanoparticles for Multimodality Therapy of Tumors
Source: Adv Sci (Weinh). 2021 Jul 8;8(17):2100074. doi: 10.1002/advs.202100074 (PMC8425924; doi:10.1002/advs.202100074)
Supplement: Supplementary file 1 — Supporting Information [file ADVS-8-2100074-s001.pdf]

© 2021 Wiley-VCH GmbH

ADVANCED SCIENCE Open Access

# Supporting Information

for *Adv. Sci.*, DOI: 10.1002/advs.202100074

# HOCl-Activated Aggregation of Gold Nanoparticles for Multimodality Therapy of Tumors

*Dongya Liu, Lingyan Liu, Feiyang Liu, Mengfan Zhang, Peng Wei,* and Tao Yi**

# 1. Experimental Section

## 1.1 Chemicals and Materials

$HAuCl_4·3H_2O$,sodium citrate,DL-lipoic acidand 14.5% NaOCl solution werepurchased fromJ&K Scientific Ltd(Beijing, China). PEG-SH with a molecularweight of 5000 kDa was obtained from Yare Biotech (Shanghai, China).Dulbecco's modified Eagle's medium (DMEM)andfetal bovine serum (FBS)were purchased from Thermo Fisher Scientific.1, 3-diphenylisobenzofuran (DPBF) was purchased from Macklin Biochemical (Shanghai). HATUand N,N-diisopropylethylamine (DIPEA) were bought from Aladdin Reagent Co. Ltd.(Shanghai, China).Methythiazolyl tetrazolium (MTT) and cell culture reagents were purchased from Beyotime or Invitrogen. All starting materials were obtained from commercial suppliers and used as received.General chemicals were purchased fromJ&K China Chemical Ltd, Energy Chemical, Adamas-beta®, Sigma-Aldrich, Dojindo and Sinopharm Chemical Reagent Co., Ltd.

All experiments were performed in compliance with therelevant laws and institutional guidelines. All animal experiments were performed according to procedures approved by theFudan University Committee on Animal Care and Use.

## 1.2 Instrumentation and characterization

$^1H$ NMR and $^{13}C$ NMR were carried out on a Bruker Avance 400 spectrometer with tetramethylsilane (δ = 0.0 ppm) or the solvent residue peak $CDCl_3$ (7.26 ppm for $^1H$, 77.16 ppm for $^{13}C$), $CD_3CN$ (1.94 ppm for $^1H$, 1.32 ppm for $^{13}C$), Acetone-$d_6$ (2.05 ppm for $^1H$, 29.84 ppm for $^{13}C$) or DMSO-$d_6$ (2.50 ppm for $^1H$, 39.52 ppm for $^{13}C$) as an

internal standard (Bruker, Switzerland). High-resolution mass spectra were recordedon a VG ZAB-HS mass spectrometer (VG Instruments, UK).Fourier transform infrared spectroscopy(FT-IR) spectra were carried out on a Nicolet iS10 spectrometer (Thermo Fisher Scientific, USA) using a KBr pellet.The absorption spectra were performed on a UV 3600 UV-vis-NIRspectrophotometer (Shimadzu Co., Japan). The fluorescence spectra were acquired on an F-4500spectrofluorometer (Hitachi, Japan).The transmission electron microscopy (TEM) images wereperformed on a Tecnai G2 F20 field emission transmission electron microscope (FEI, US) with anaccelerating voltage of 200 KV. Dynamic light scattering (DLS) and zeta potential analysis wereperformed on a Nano-ZS Zetasizer (Malvern, UK).MTT assay wasperformed on a microplate reader (Synergy HT, BioTek).Photoacoustic imaging was performed using Vevo LAZR multi-mode photoacoustic/ultrasonic imaging system with YAG laser, optical parametric oscillator (OPO) (FujiFilm VisualSonics Inc.).Thermal imagingwas recorded bya thermal camera(FLIR) and the quantification analysis was performed by the FLIR Examiner software.

**1.3 Synthesis of FDOCl-23**

FDOCl-23was prepared according to our published procedures. The synthetic route is given as follows.

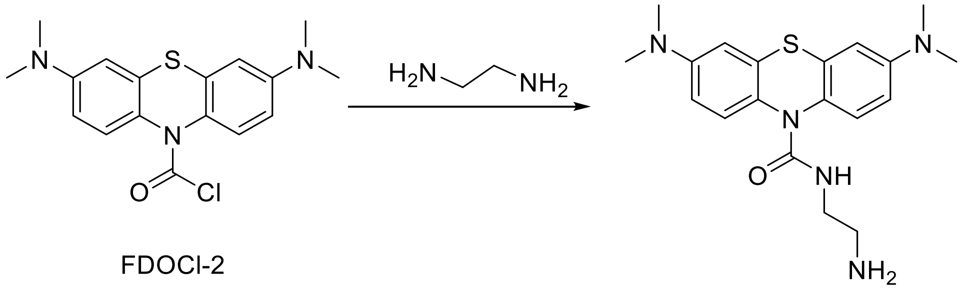


FDOCl-2

FDOCl-23

FDOCl-2 (1.0 g, 2.87 mmol, 1.0 eq), $Na_2CO_3$ (0.91 g, 8.61 mmol, 3.0 eq), were dissolved in 10 mL of dichloromethane, the resultingmixture was stirred in an ice-water bath. Ethanediamine (11.48 mmol, 4.0 eq) in dichloromethane were added dropwise. After addition,the mixture was stirred at room temperature until the reaction completed as indicated by TLC analysis which was conducted at 1 hintervals.The reaction mixture was poured into 200 mL of ice-water while stirring, and the resulting mixture was extracted with 3 × 150 mLportions of ethyl acetate. The combined extracts were washed with brine, dried over anhydrous sodium sulfate and evaporated on arotary evaporator to afford an oily residue, which was purified by column chromatography (ethyl acetate/petroleum ether = 1/5) to yield FDOCl-23as a light green solid (yield 40%).

$^1$H NMR (400 MHz, DMSO-$d_6$) δ 7.28 (d, $J$ = 8.8 Hz, 2H), 6.70 (d, $J$ = 2.8 Hz, 2H), 6.67 – 6.64 (m, 2H), 6.00 (t, $J$ = 5.4 Hz, 1H), 3.01 (q, $J$ = 6.0 Hz, 2H), 2.89 (s, 12H), 2.55 (t, $J$ = 6.2 Hz, 2H), 1.37 (s, 2H).

$^{13}$C NMR (100 MHz, DMSO-$d_6$) δ 155.7, 148.9, 133.6, 128.9, 127.8, 111.6, 110.8, 43.89, 41.8, 40.7.

HRMS (ESI Positive): calc. for $C_{19}H_{25}N_5OS$ $[M+H]^+$372.1853, found 372.1858.

**1.4 Synthesis of FDOCl-24**

The synthetic route is given as follows:

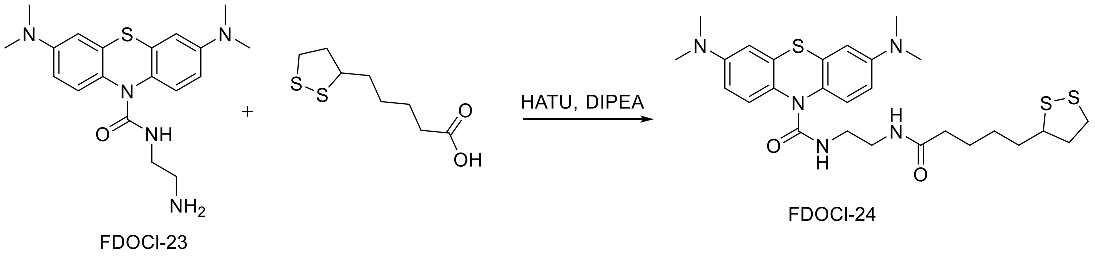

FDOCl-24was synthesized by condensation reaction between FDOCl-23and DL-lipoic acid. DL-lipoic acid (0.82 g), FDOCl-23 (0.455 g), HATU(1.26 g) and DIPEA (0.57 g) (with a molar ratio of 1:1:1.5:2) were dissolved in DMSO. The mixture was stirred and incubated at room temperature for 24 h to giveFDOCl-24as a light yellow solid (yield 70%).

$^{1}$H NMR (400 MHz, DMSO-$d_6$) δ 7.76 (s, 1H), 7.28 (d, $J$ = 8.8 Hz, 2H), 6.70 (d, $J$ = 2.8 Hz, 2H), 6.65 (dd, $J$ = 8.8, 2.8 Hz, 2H), 6.09 (s, 1H), 3.63 – 3.56 (m, 1H), 3.21 – 3.06 (m, 6H), 2.89 (s, 12H), 2.43 – 2.36 (m, 1H), 2.05 (t, $J$ = 7.4 Hz, 2H), 1.89 – 1.81 (m, 1H), 1.70 – 1.61 (m, 1H), 1.59 – 1.47 (m, 3H), 1.37 – 1.32 (m, 2H).

$^{13}$C NMR (100 MHz, DMSO-$d_6$) δ 172.2, 155.2, 148.5, 133.1, 128.4, 127.3, 111.1, 110.3, 56.1, 40.2, 40.1, 39.9, 38.6, 38.1, 35.3, 34.2, 28.4, 25.0.

HRMS (ESI Positive): calc. for $C_{27}H_{37}N_5O_2S_3$ $[M+H]^+$ 560.2182, found 560.2198.

**1.5 Preparation of HOCl-responsive Au-MB-PEG NPs**

AuNPswere prepared by a typical citrate reduction method with slight modification.[1] Briefly, 100 mL of 0.01% (w/v) $HAuCl_4$ solution was heated to boiling and 2.7 mL of 1% (w/v) sodium citrate solution was quickly added under constant stirring, and maintained at the boiling temperature for an additional 15 min. Then the reaction was terminated by cooling the reaction system down to room temperature. The resulting AuNP solution was stored at 4 °C for further experiments.The concentration of the obtained AuNP solution was calculated at 1.17 nmol/L according to the reportedmethod.[1]

HOCl-responsive Au-MB-PEG NPs were prepared by the following

procedure:the as-synthesized citratestabilized AuNPs (2.34 nmol/L,5 mL) wascentrifugedand the supernatant was removed.The solid was thendissolved in DMF (5 mL). FDOCl-24in DMF (10 mg/mL, 20 μL) was added andthe solution was stirred for three hours.Subsequently, $mPEG_{5000}$-SH(50 μM, 50 μL)wasadded and stirred at room temperature overnight. Finally, these AuNPs were washed by centrifugation for threetimes to remove excess amount of ligands and finally dissolved in water.

**1.6*In vitro* photothermal effects of Au-MB-PEG NPs**

In a typical experiment,PBS and Au-MB-PEGNPs (0.2 mg/mL)in the absence or presence of 10 μM HOCl were irradiated with an 808 nm laser at power density of 1.5 $W/cm^2$for 10 min. Different concentrations of the AuNPs aggregates (0.2, 0.4, 0.6, 0.8 and 1 mg/mL) were also irradiated with an 808 nm laser at the same power densityfor 10 min. The temperature of the solutions was continuously monitored with athermal camera.

The photothermal conversion efficiency was calculated with the following equation according to a previously reported method.

$$\eta = \frac{mc(T_{\max} - T_s)}{I(1 - 10^{-A})\tau_s}$$

where $m$ is the solution mass, $c$ is the heat capacity of water (4.2 J/g), $T_{max}$ and $T_s$ are maximum temperatures achieved in the presence or absence of the aggregated AuNPs. $I$ is the laser power density (1.5 $W/cm^2$), $A$ is the absorbance of the aggregated AuNPs solution at 808 nm, and $\tau_s$ is system time constant (180 s). The photothermal conversion efficiency ($\eta$) of Au-MB-PEG NPs is 20%.

**1.7*In vitro*singlet oxygen ($^1O_2$) generation of Au-MB-PEG NPs**

The DPBF was used for evaluatingthe$^1O_2$ produceability of Au-MB-PEG NPs, whichreacted with$^1O_2$ to cause a decrease in the absorption at 410 nm. Briefly, Au-MB-PEG NPs solution containing DPBF in the absence or presence of HOCl were irradiated with 655 nm laser (0.4 W/cm$^2$) for different periods of timeand the absorbance at 410 nm was measured by UV-Vis absorption.

**1.8*In vitro* photoacoustic imaging**

For *in vitro* PA imaging, different wavelengths (680-970 nm) of excitation light were used to collect the PA signals, from which an optimized excitation light of 680 nm was selected for the following PA imaging test.Different concentrations of HOClwere added to the Au-MB-PEG NPs solutions for PA signal detection and the linearity evaluation of the PA signal.

**1.9*In vitro* cytotoxicity assay**

HepG2 cells were treated with high-glucose Dulbecco's modified Eagle's medium (DMEM) containing 10% fetal bovine serum and 1% penicillin-streptomycin and were incubated under 37°Cwithin a humidified atmosphere of 5% $CO_2$. HepG2 cells were seeded onto a 96-well plate at a density of $10^{4/}$well and cultured for 24 h to obtain monolayer cells.Then, different concentrations ofAu-MB-PEG NPs (0, 0.1, 0.2, 0.3, 0.4, 0.5, 0.6, 0.7 and 0.8 mg/mL)in DMEM media were added into the cell and co-incubated for 12 or 24 h. To assess cell viability, 100 μL 1 mg/L MTT were added into each well. After incubation for 4 h, MTT solution was removed, 150 μL DMSO were added to the wells and the absorbance at 490 nm was recorded via a microplate reader. Cell viability was calculated as thesurvival percentage of control.

**1.10*In vivo* photoacoustic and thermal imaging**

For PA imaging, the HepG2 tumor-bearing nude mice weredivided into two groups and subjected to intravenous injection of Au-PEGand Au-MB-PEGNPs (2mg/mL, 200 μL), respectively. And PA imaging were recorded at 0, 2, 4, 6, 12, 24 and 48 h postinjection. For thermal imaging, PBS, Au-PEGand Au-MB-PEGNPs treated mice (12 h postinjection) was recorded by an infrared camera (FLIR) when the tumors were exposed to 808 nm laser (1.5 $W/cm^{-2}$) for 10 min.

**1.11*In vivo* synergisticphotothermal and photodynamic therapy**

The HepG2 tumor-bearing nude micewere randomly allocated into six groups (4 mice per group) before the experiments: (1) injection with PBS as the control group; (2) injection with PBS followed by laser irradiation (laser: 808+ 655 nm); (3) injection with Au-PEG followed by laser irradiation (808 +655 nm); (4) injection with Au-MB-PEG by PDT only (655 nm); (5) injection with Au-MB-PEG by PTT only (808 nm);(6) intravenous injection with Au-MB-PEG followed by laser irradiation (808+655 nm).The power density of 808 nm laser for PTT was 1.5 $W/cm^{-2}$ and the exposure time was10 min.The power density of 655 nm laser for PDT was 0.4 $W/\ cm^{-2}$ and the exposure time was10 min.The photothermal and photodynamic treatment was performed at 12 hpostinjection. Tumor size was measured with a caliper every 2 days and tumor volume wascalculated with the following equation: $V = ab^2/2$, where $V$ is the volumeof the tumor, and $a$ and $b$ are tumor length and tumor width, respectively.The tumor volume and body weight was continuously monitored till 14-day post-treatment.The tumor tissues were resected from the mice after 3-day treatmentfor

evaluating the therapeutic efficacy of different treatments throughpathological analysis. The mice were sacrificed and the main organs including heart, liver, spleen, lung and kidney were collected and fixed with 4% formaldehyde for hematoxylin and eosin (H&E) staining.

**1.12 Blood routine analysis of Au-MB-PEG NPs**

The healthy mice were i.v.injected with Au-MB-PEG NPs(2 mg/mL, 200 μL) and the blood was collected at time pointof one week post-injection. Blood routine (white blood cells (WBC), platelets (PLT), platelet larger cell count (P-LCC), hemoglobin (HGB), mean corpuscular hemoglobin concentration (MCHC), Hematocrit (HCT),coefficient variation of red blood cell volume distribution width (RDW-CV),thrombocytocrit (PCT), mean corpuscular volume (MCV), standard deviation in red cell distribution width (RDW-SD), mean platelet volume (MPV), blood cells (RBC), mean corpuscular hemoglobin (MCH), platelet distribution width (PDW)) were thenmeasured by semiautomatic biochemistry analyzer.

**1.13 Statistical Analysis**

Data are shown as mean ± s.d. of triplicates unlessotherwise indicated. Statistical analysis was performed using a two-tailedStudent's t-test. $P < 0.05$ was considered statistically significant, and $P< 0.01$ was considered highly statistically significant. Statistical analysiswas performed with Microsoft Excel software. No animals were excludedfrom the analysis.

## 2. Additional Figures and Images

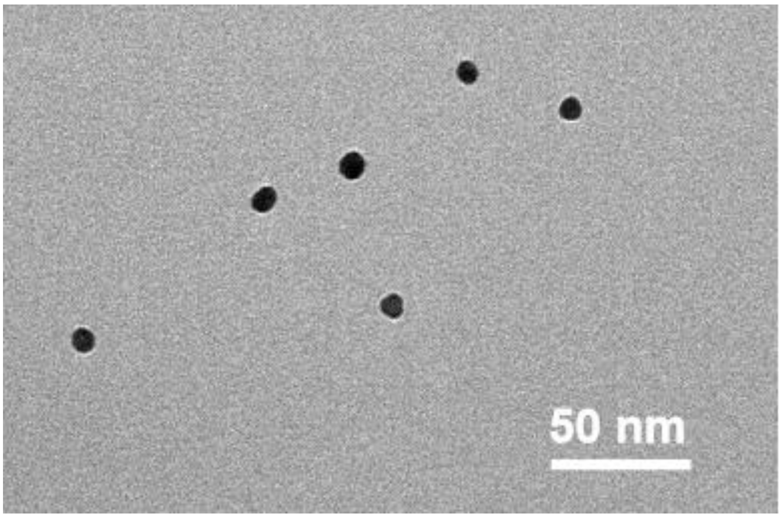


**Figure S1**. TEM of citrate-cappedAuNPs.

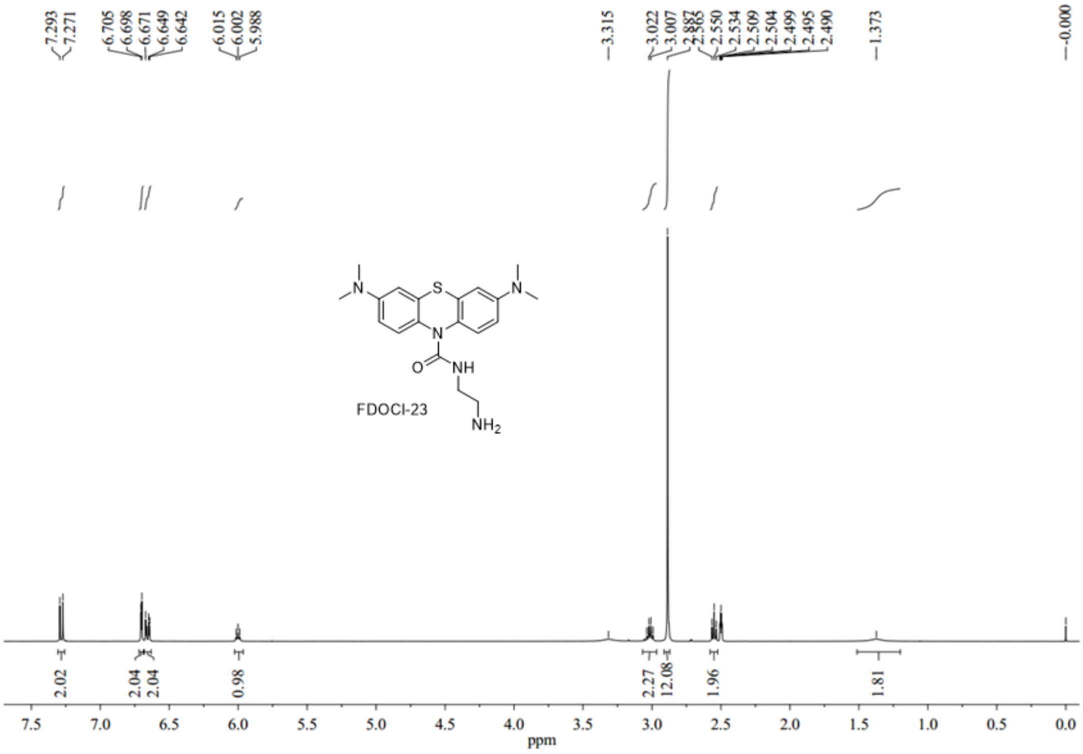


**Figure S2**.$^{1}$HNMR spectra of FDOCl-23.

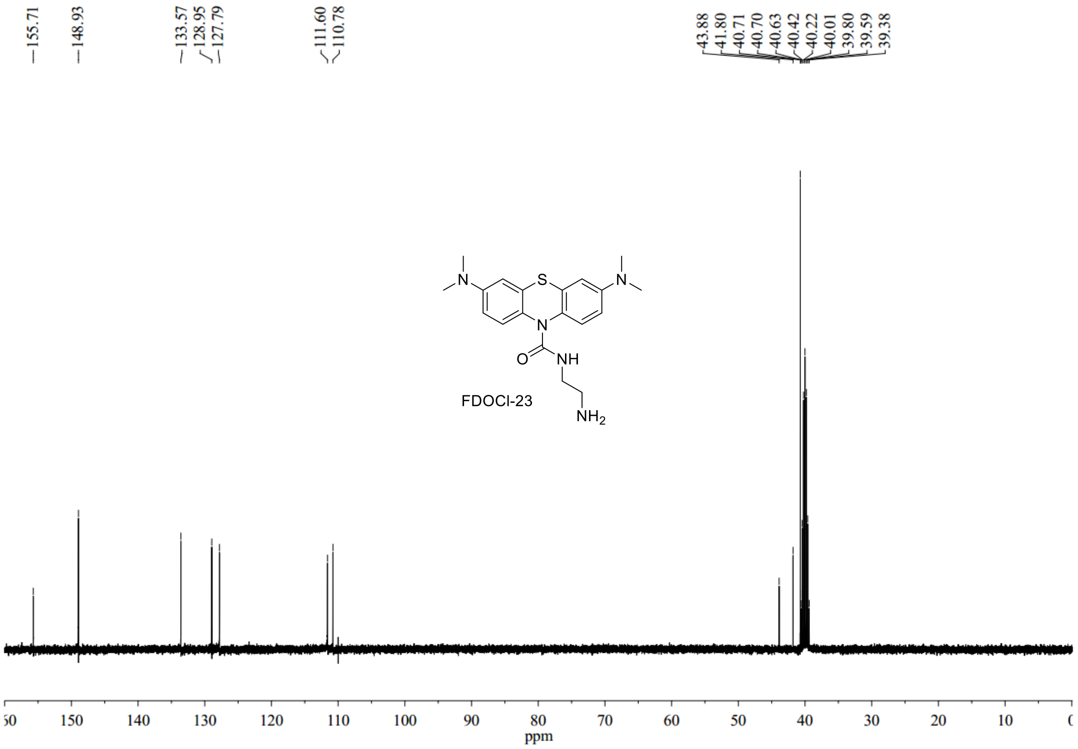


**Figure S3**. $^{13}$C NMR spectraof FDOCl-23.

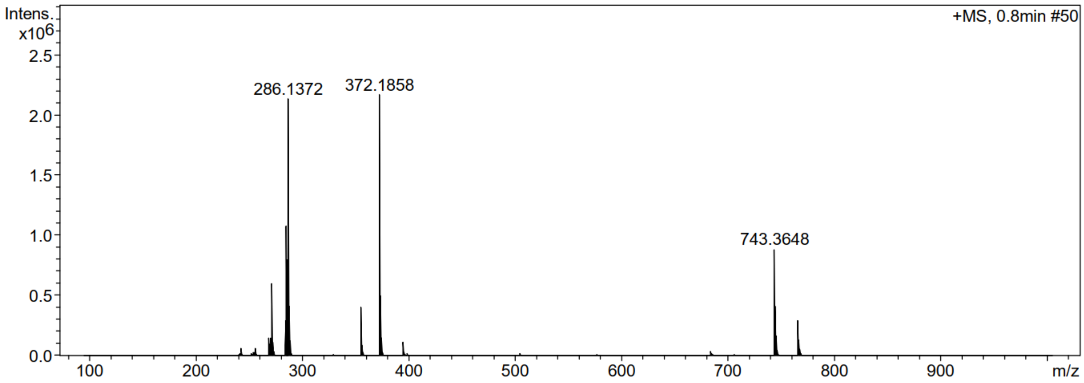


**Figure S4**. HRMS spectrum of compound FDOCl-23.

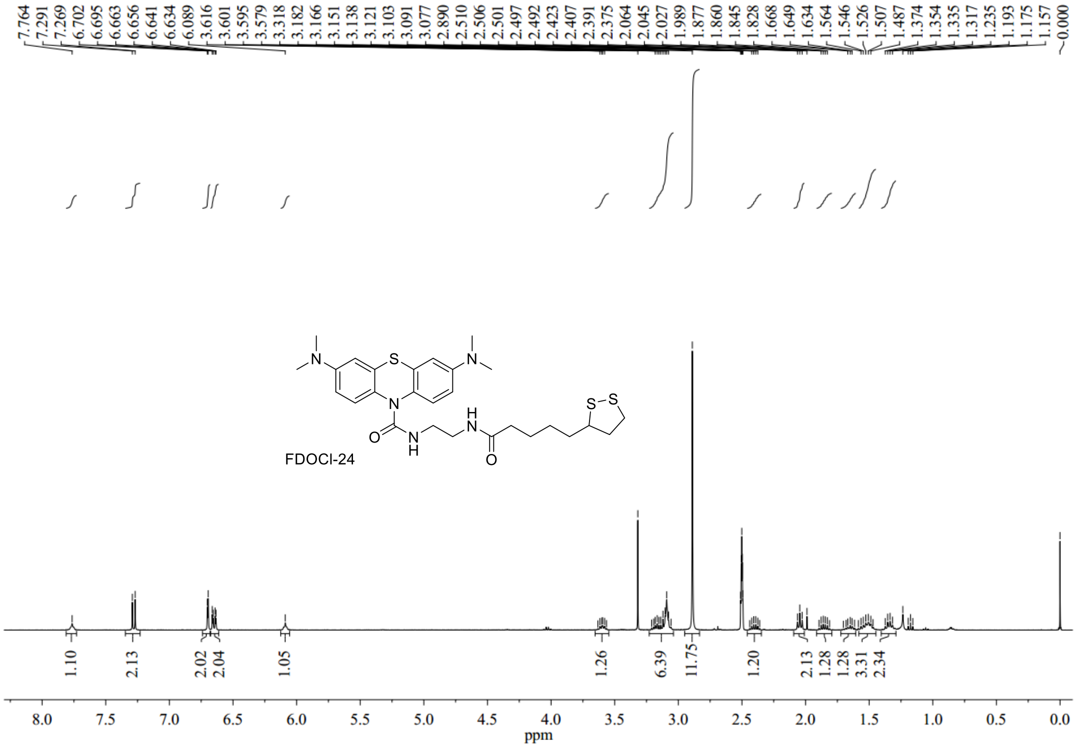

**Figure S5**.$^{1}$HNMR spectra of FDOCl-24.

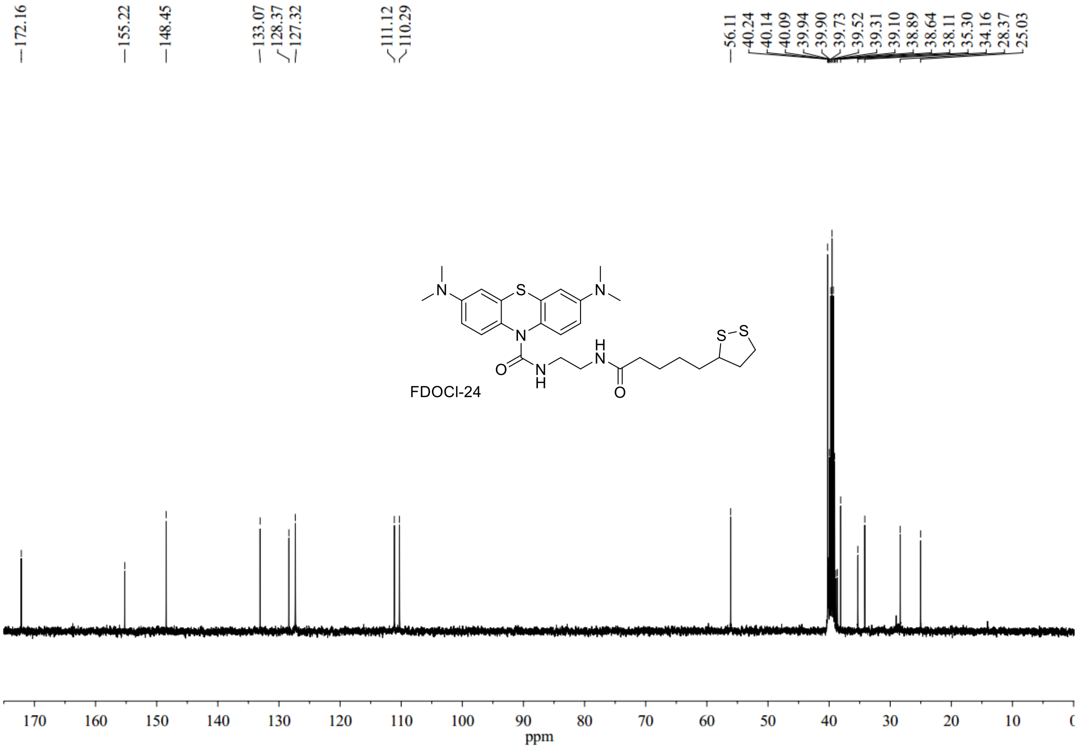

**Figure S6**. $^{13}$C NMR spectraof FDOCl-24.

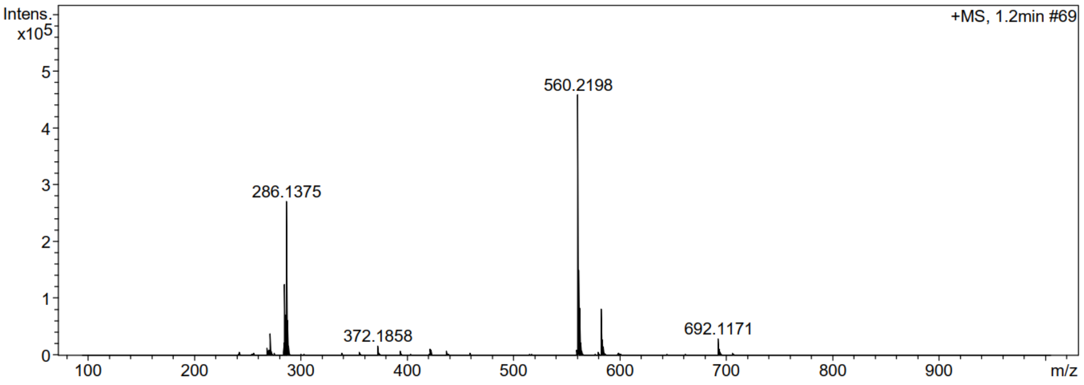


**Figure S7**. HRMS spectrum of FDOCl-24.

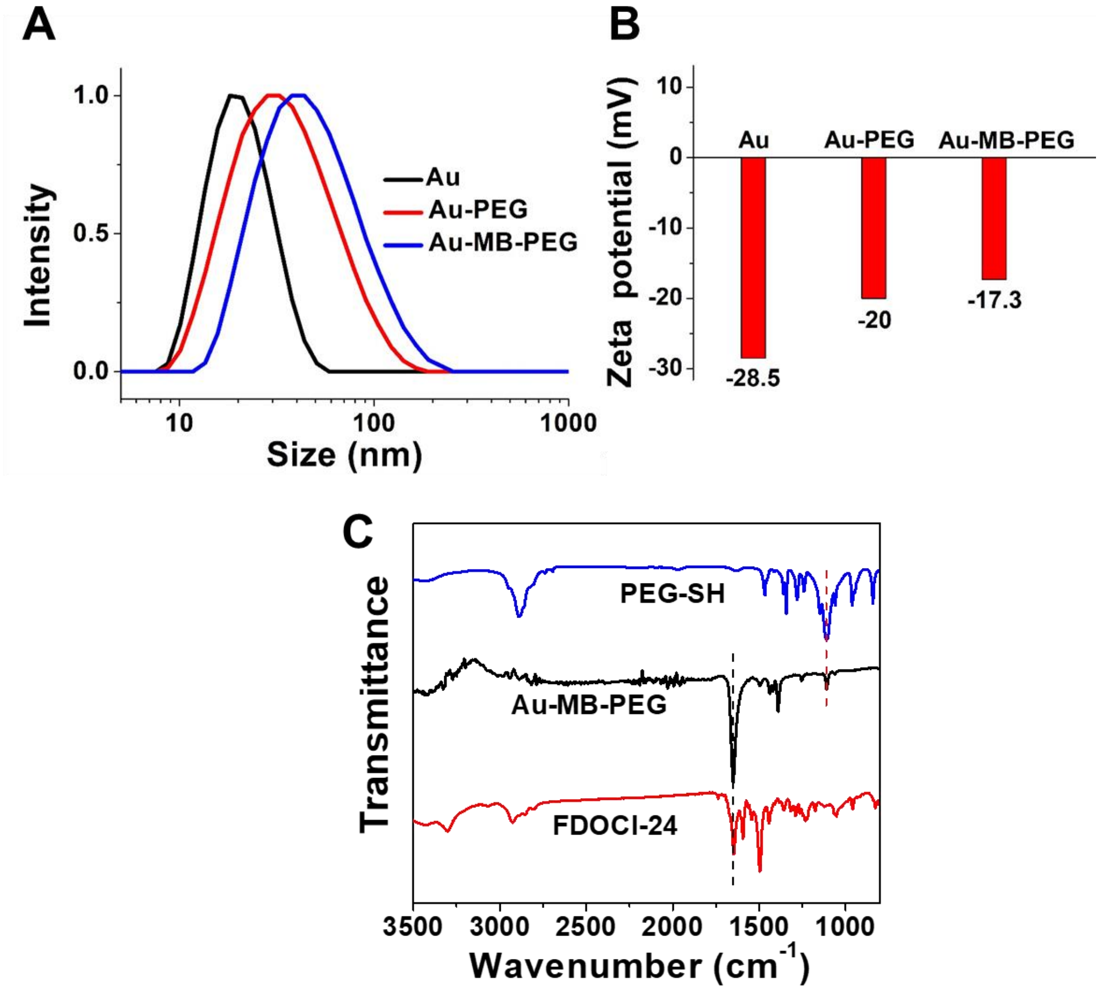


**Figure S8**. Characterization ofAu-MB-PEG NPs.(A)Hydrodynamic diameter and (B)Zeta potential of AuNPs, Au-PEG NPs and Au-MB-PEG NPs; (C)FT-IR spectraofAu-MB-PEG NPs.

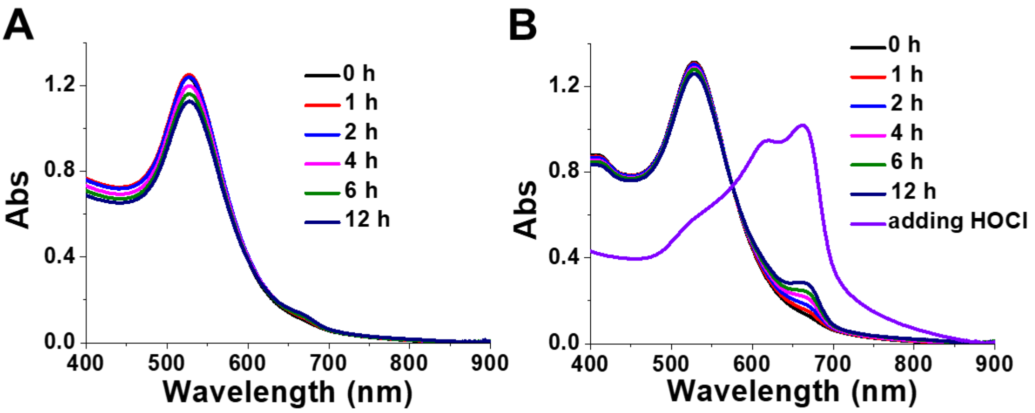


**Figure S9**. The stability of Au-MB-PEG NPs (A)in aqueous solutionand (B)DMEM supplemented with 10% fetal bovine serum (FBS).Comparison of absorption spectra of Au-MB-PEG NPs in culture medium and aggregated Au-MB-PEG NPs after adding HOCl.

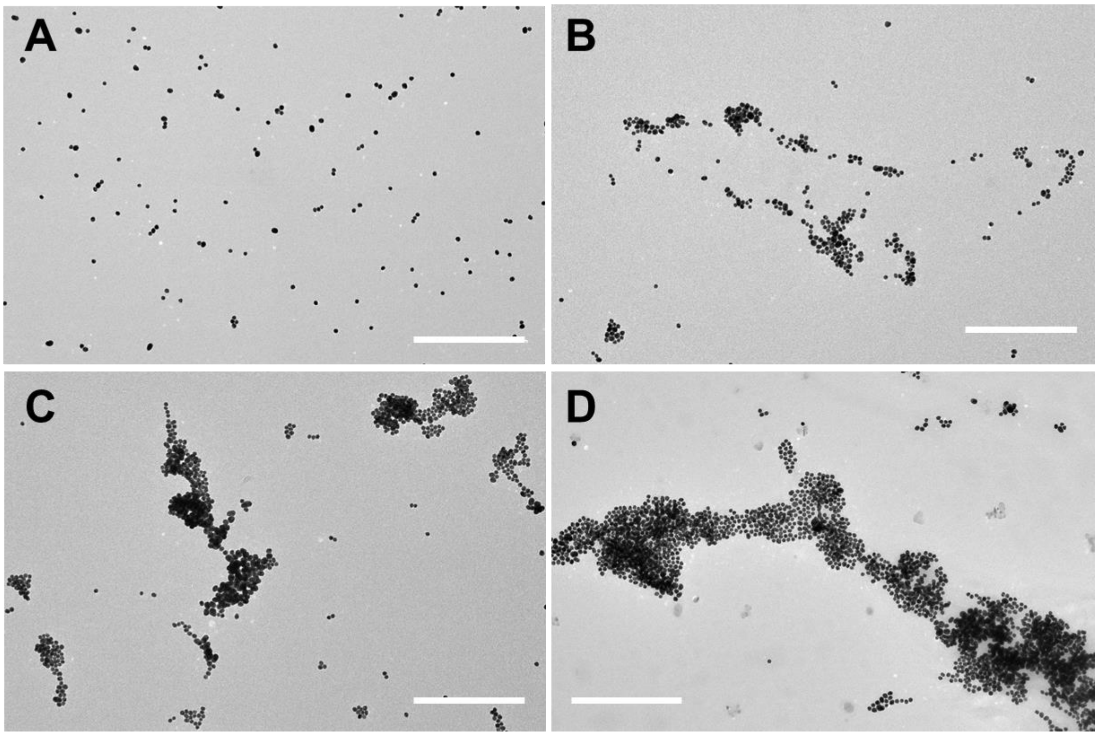


**Figure S10**. Aggregation characterization of Au-MB-PEG NPs. TEM images of (A)Au-MB-PEG NPs only, (B-D) with 5 μM, 10 μM and 20 μMHOCl, respectively. The scale bar is 500 nm.

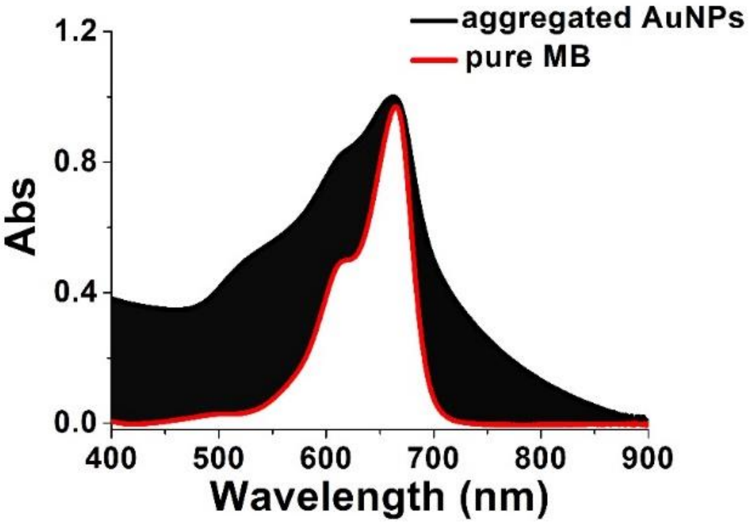


**Figure S11**. The superimposed UV-Vis spectra of aggregated AuNPs and pure MB.

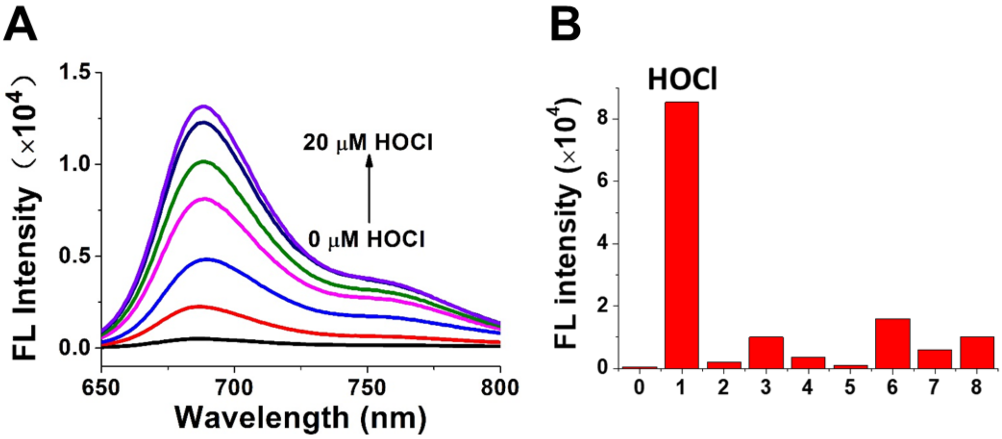


**Figure S12**.(A) Fluorescent spectra of Au-MB-PEG NPs before/after addition of different concentration of HOCl; (B) Selectivity of FDOCl-24 (5 μM) toward HOCl (5 μM) and other ROS (25 μM) (from 0 to 8:blank, HOCl, $H_2O_2$,•OH, $O^{2-}$,t-BuOOH, t-BuOO•, NO, $ONOO^-$).

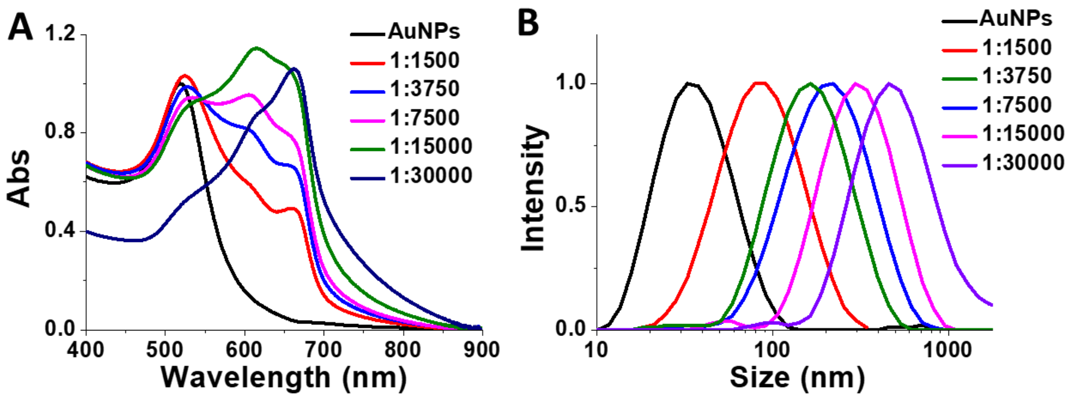


**Figure S13**. Absorption spectra (A) and hydrodynamic diameter (B) of Au-MB-PEG NPs from varied molar ratio of AuNPs and FDOCl-24 after reaction with the same concentration of HOCl (10 μM).

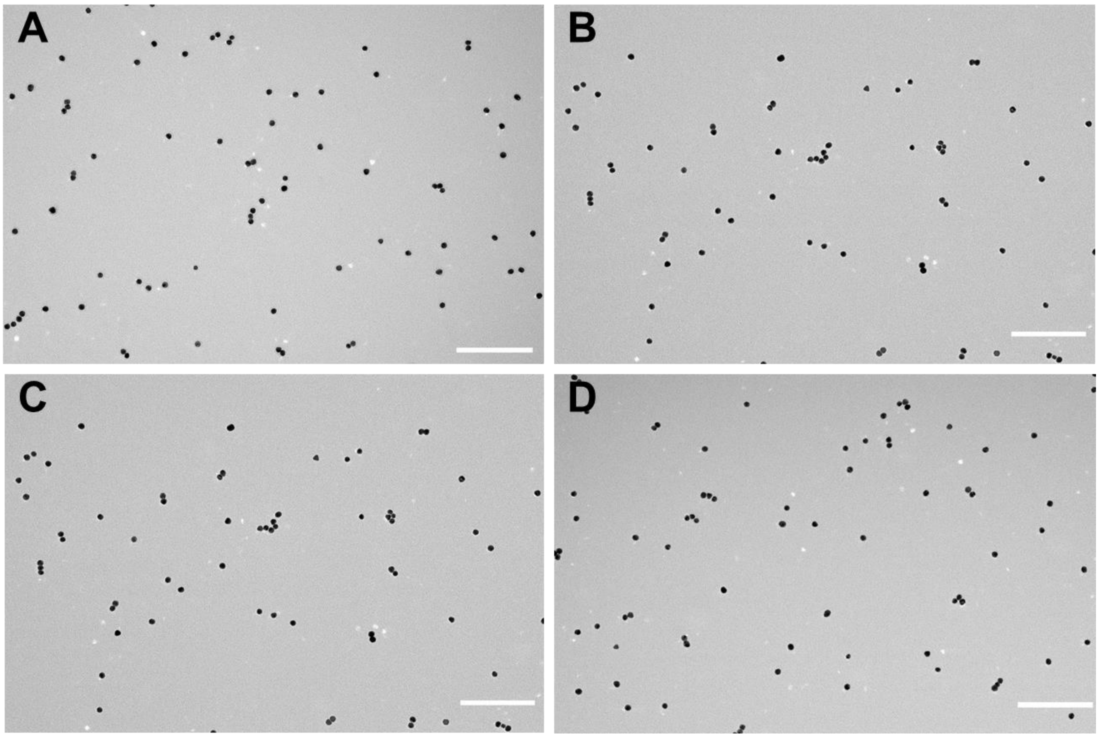


**Figure S14**. TEM images of (A)Au-PEG NPs only, (B-D) with 5 μM, 10 μM and 20 μMHOCl, respectively. The scale bar is 200 nm.

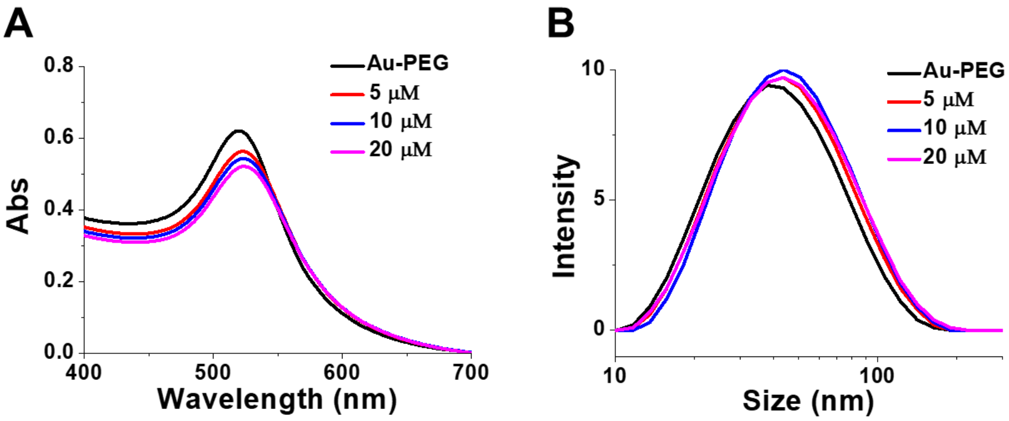


**Figure S15**.UV-Vis absorption (A) and hydrodynamic diameter (B) of Au-PEG NPs in presence of increasing concentration of HOCl.

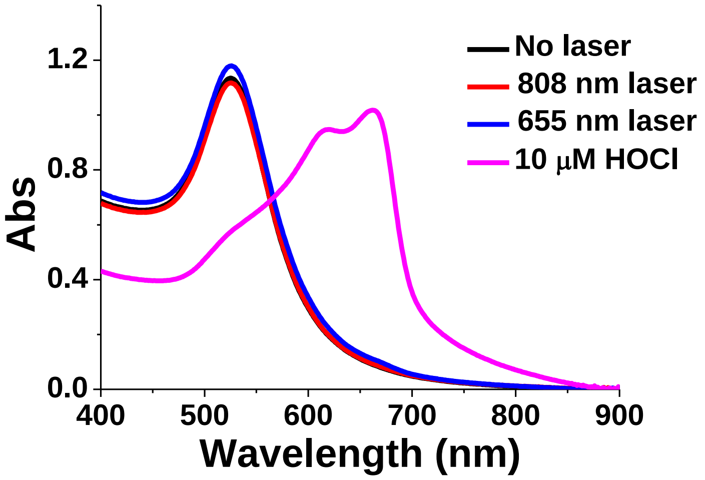


**Figure S16**. The absorption spectra of Au-MB-PEG NPs irradiated by 808 (1.5 W/cm$^2$) and 655 nm (0.4 W/cm$^2$) laser for 30 min and then incubated with 10 μMHOCl.

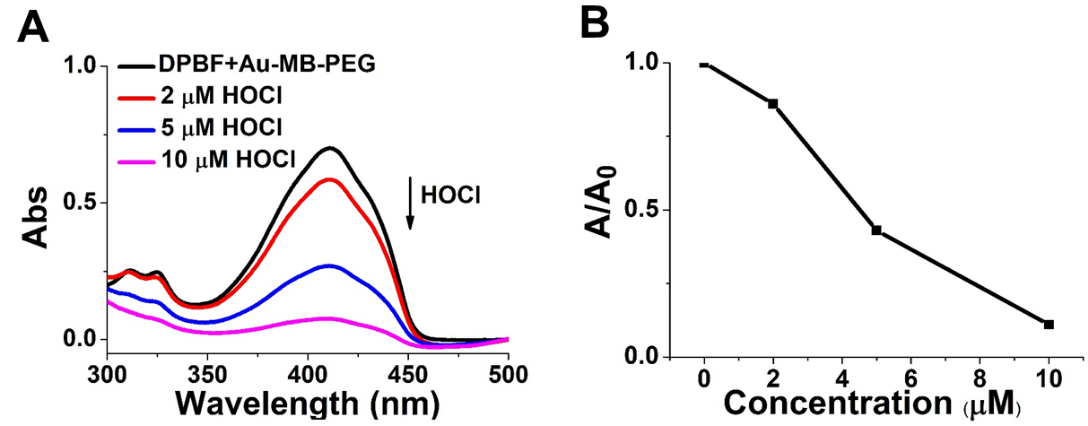


**Figure S17**. In vitro singlet oxygen ($^1O_2$) generation of Au-MB-PEG NPs. (A) UV−Vis spectra of DPBF containing Au-MB-PEG NPs with increasing HOCl concentrations and (B) corresponding relative absorption intensity decay under 655 nm laser irradiation (0.4 W/cm$^2$, 10 min).

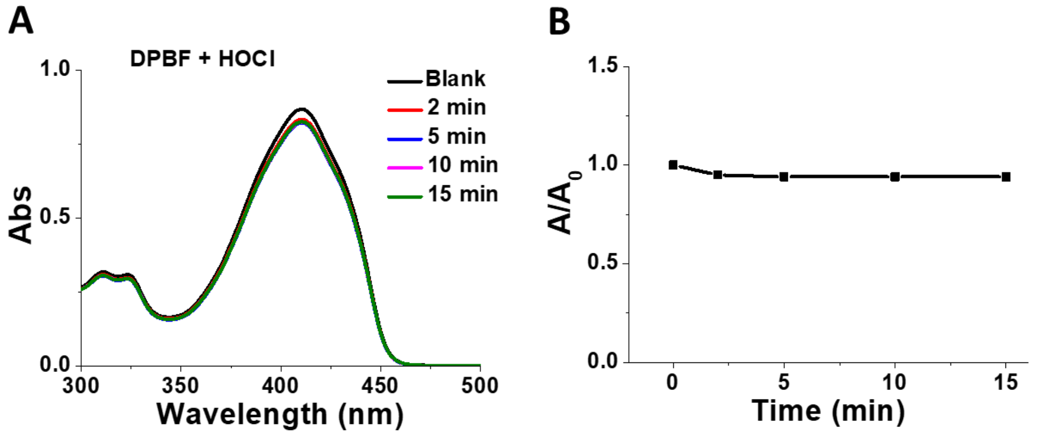


**Figure S18**. (A) UV−Vis spectra of DPBF containing 10 μM HOCl and (B) corresponding relative absorption intensity variations within 15 min.

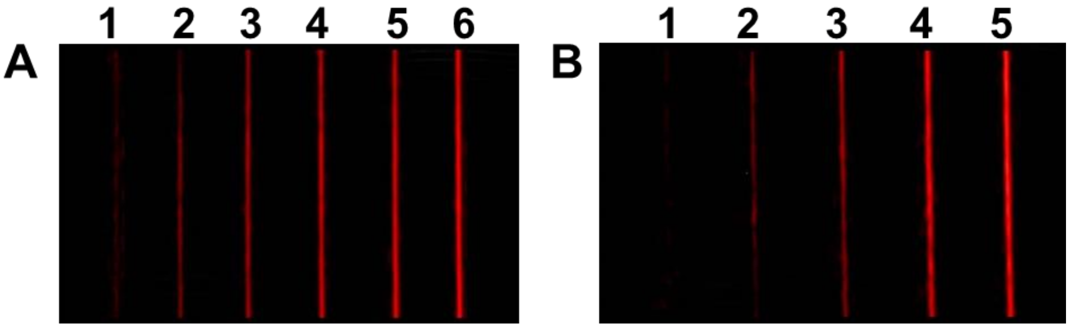


**Figure S19**. (A) PA images of Au-MB-PEG NPs reacting with different HOCl concentrations (1-6: 0, 2, 5, 10, 15, 20 μMHOCl); (B) PA images of self-assembled Au-MB-PEG NPs at different concentrations (1-5: 0.2, 0.4, 0.6, 0.8, 1 mg/mL).

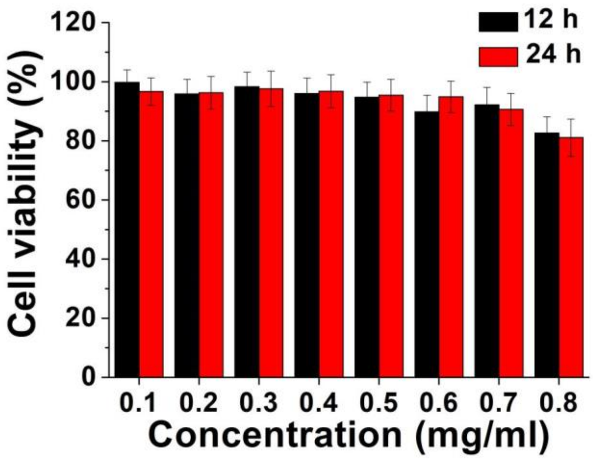


**Figure S20**. Viability of HepG2 cells after incubated with differentconcentrations of Au-MB-PEG NPs for 12 h and 24 h, respectively. Data are presented as means ± s.d. (n = 5).

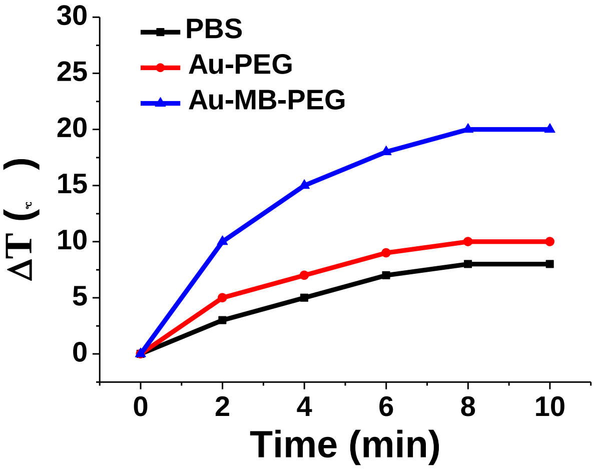


**Figure S21**.Tumor localtemperature changes against irradiation time of808nm laser after treatment with PBS,Au-PEG, and Au-MB-PEG NPsat 12h postinjection.

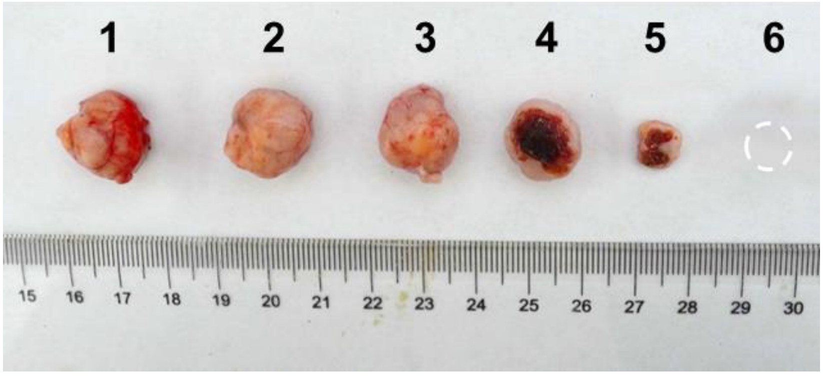


**Figure S22**. Photographs of tumors harvested on 14th day post-treatments from micereceiving different treatments. Group1-6: PBS,PBS+Laser (808+655 nm), Au-PEG+Laser (808+655 nm),Au-MB-PEG+Laser (655 nm), Au-MB-PEG+Laser(808 nm), Au-MB-PEG + Laser (808+655 nm).

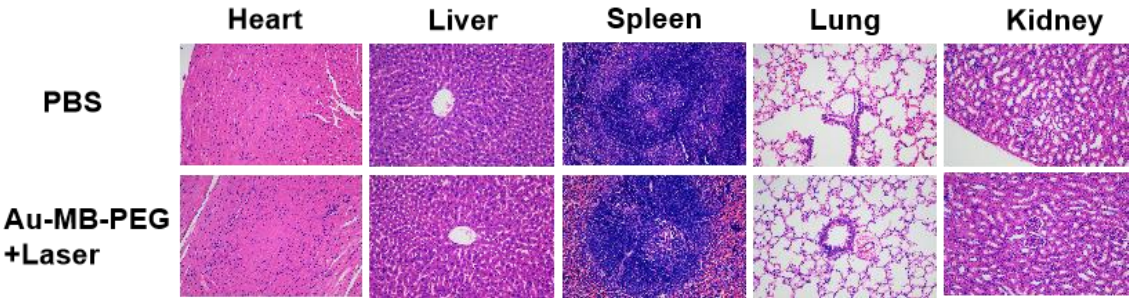


**Figure S23**. H&E staining of main organs of mice after different treatment.

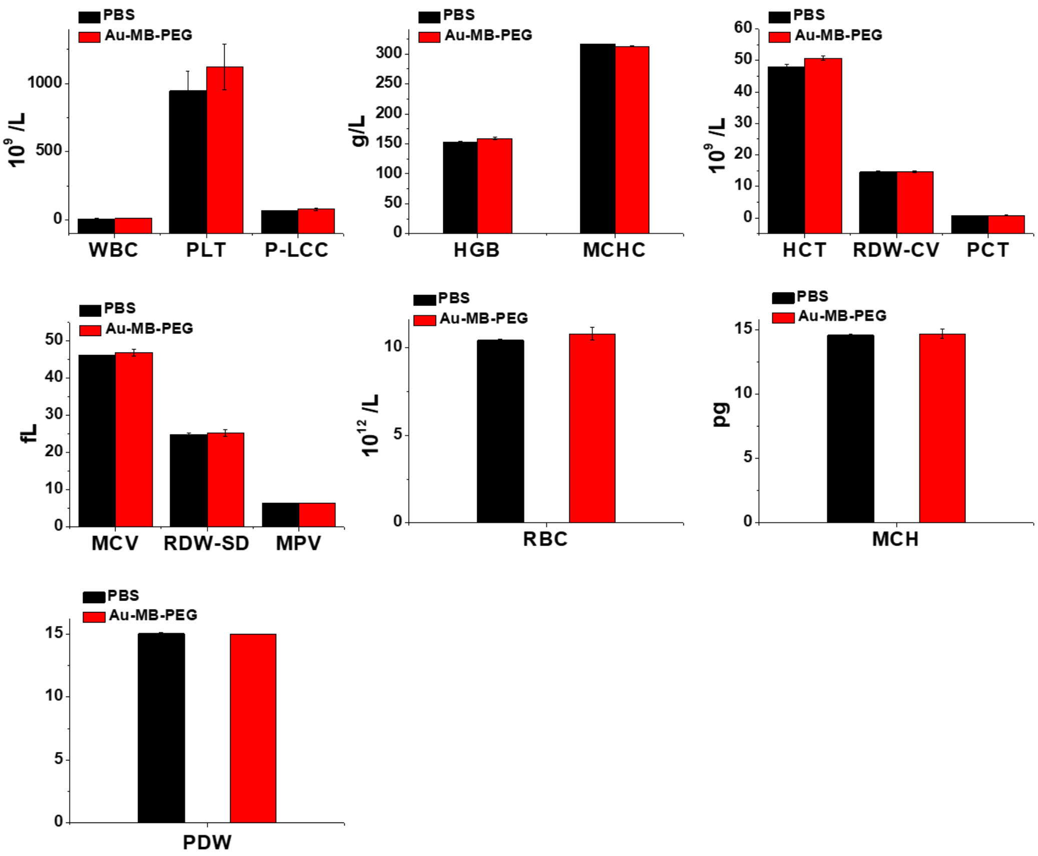


**Figure S24**. Blood biochemistry and hematology analysis of from BALB/c mice in 7 days after injection with PBS and Au-MB-PEG. Shown are the white blood cells (WBC), platelets (PLT), platelet larger cell count (P-LCC), hemoglobin (HGB), mean corpuscular hemoglobin concentration (MCHC), Hematocrit (HCT),coefficient variation of red blood cell volume distribution width (RDW-CV),thrombocytocrit (PCT), mean corpuscular volume (MCV), standard deviation in red cell distribution width (RDW-SD), mean platelet volume (MPV), blood cells (RBC), mean corpuscular hemoglobin (MCH), platelet distribution width (PDW).Data are presented as means ± s.d. (n = 3).

# 3 Additional Table

**Table S1.**Reported stimuli-responsive aggregation nanoparticles

| stimuli | material | responsive time | aggregationmechanism | Ref. |
|---|---|---|---|---|
| | AuNPs | 12 h | click reaction | [2,3] |
| enzyme | | 2h | DNA hybridization | [4] |
| | | 24h | IntermolecularH-bonds. | [5] |
| | UCNPs | 4 h | click reaction | [6] |
| pH | AuNPs | 90 min | Electrostaticinteractions | [7] |
| | | 60 min | Electrostaticinteractions | [8] |
| GSH | $Fe_3O_4$ | 5 h | click reaction | [9] |
| $H_2O_2$ | AuNPs | 4h | Hydrophobic interactions | [10] |
| light | AuNPs | 25 min | C−C, C−H, O−H, X−H insertions | [11] |

## Reference:


[1] J. Li, H. Duan, P. Xu, X. Huang, Y. Xiong,*RSC Adv.***2016**, *6*, 26178.

[2] S. Ruan, C. Hu, X. Tang, X. Cun, W. Xiao, K. Shi, Q. He, H. Gao, *ACS Nano***2016**, *10*, 10086.

[3] Q. Hu, W. Sun, Y. Lu, H. N. Bomba, Y. Ye, T. Jiang, A. J. Isaacson, Z. Gu, *Nano Lett.***2016**, *16*, 1118.

[4] K. Yang, Y. Liu, Y. Wang, Q. Ren, H. Guo, J. B. Matson, X. Chen, Z. Nie, *Biomaterials***2019**, *223*, 119460.

[5] S. Yang, D. Yao, Y. Wang, W. Yang, B. Zhang, D. Wang, *Chem. Commun.***2018**, *54*, 9841.

[6] X. Ai, C. J. H. Ho, J. Aw, A. B. E. Attia, J. Mu, Y. Wang, X. Wang, Y. Wang, X. Liu, H. Chen, *Nat. Commun.***2016**, *7*, 1.

[7] J. Nam, N. Won, H. Jin, H. Chung, S. Kim, *J. Am. Chem. Soc.***2009**, *131*, 13639.

[8] R. Zhang, L. Wang, X. Wang, Q. Jia, Z. Chen, Z. Yang, R. Ji, J. Tian, Z. Wang, *Adv. Healthc. Mater.***2020**, *9*, 2000394.

[9] Z. Gao, Y. Hou, J. Zeng, L. Chen, C. Liu, W. Yang, M. Gao, *Adv. Mater.***2017**, *29*, 1701095.

[10] S. Wu, S. Y. Tan, C. Y. Ang, Z. Luo, Y. Zhao,*Chem. Commun.***2016**, *52*, 3508.

[11] X. Cheng, R. Sun, L. Yin, Z. Chai, H. Shi, M. Gao, *Adv. Mater.* **2017**, *29*, 1604894.
